# Concomitant memantine and *Lactobacillus plantarum* treatment attenuates cognitive impairments in APP/PS1 mice

**DOI:** 10.18632/aging.102645

**Published:** 2020-01-06

**Authors:** Qiu-Jun Wang, Yue-E Shen, Xin Wang, Shuang Fu, Xin Zhang, Yi-Na Zhang, Rui-Tao Wang

**Affiliations:** 1 General Practice Department, The Second Affiliated Hospital, Harbin Medical University, Harbin 150086, China; 2 Department of Neurology, The First Affiliated Hospital of Harbin Medical University, Harbin 150001, China; 3 Department of Internal Medicine, Harbin Medical University Cancer Hospital, Harbin Medical University, Harbin 150081, China; 4 Department of Geriatrics, The Second Affiliated Hospital, Harbin Medical University, Harbin 150086, China

**Keywords:** Alzheimer’s disease, APP/PS1 mice, trimethylamine-N-oxide, cognitive behavior, *L. plantarum*

## Abstract

Trimethylamine-N-oxide (TMAO) is a gut microbial metabolite that promotes Alzheimer’s disease (AD) progression. Given that probiotics can alleviate AD symptoms by inhibiting the synthesis of TMAO, here we investigated the correlation between TMAO and cognitive deterioration by measuring TMAO levels in the plasma of choline-treated APP/PS1 mice (an AD mouse model) with and without probiotic treatments. We found that declines in *L.* *plantarum* in the gut were associated with cognitive impairment. Moreover, 12-weeks of treatment with memantine plus *L. plantarum* ameliorated cognitive deterioration, decreased Αβ levels in the hippocampus, and protected neuronal integrity and plasticity. These effects were accompanied by reductions in TMAO synthesis and neuroinflammation. These experiments demonstrate that *L. plantarum* augments the beneficial therapeutic effects of memantine treatment in APP/PS1 mice by remodeling the intestinal microbiota, inhibiting the synthesis of TMAO, and reducing clusterin levels. Our results thus highlight intestinal microbiota as a potential therapeutic target to decrease the risk of AD.

## INTRODUCTION

The rising longevity of humans will increase the incidence of dementias such as Alzheimer’s disease (AD) [1]. Increased levels of extracellular amyloid-β (Aβ) peptide in neuritic plaques and intracellular fibrillar aggregates of phosphorylated tau are pathological hallmarks of AD in brain tissues, in addition to the widespread loss of neurons and synapses [2]. The underlying pathogeny of AD is highly complex and polyfactorial, including contributions from gut microbiota among other genetic and environmental factors [3, 4]. Elevated levels of the trimethylamine N-oxide (TMAO) metabolite are strongly associated with AD pathology [5]. Additionally, TMAO levels are elevated in cerebrospinal fluid (CSF) from AD patients compared to cognitively-unimpaired individuals and serve as an AD biomarker [6].

TMAO influences cholesterol and sterol metabolism and increased levels of it correlate with adverse cardiovascular outcomes [7]. TMAO is a gut microbial metabolite of dietary meat and fat. In the gut, unabsorbed carnitine is converted by intestinal microbiota into trimethylamine (TMA). Thereafter, TMA is oxidized to TMAO by flavin monooxygenase (FMO) in the liver [8]. By virtue of this, the intestinal flora is a major modulator of TMAO-induced diseases and thus constitutes a new potential therapeutic target [9]. Suppression of intestinal microbiota with oral broad-spectrum antibiotics attenuated TMAO-caused atherosclerosis by reducing TMAO synthesis [8]. Nevertheless, treatment with broad-spectrum antibiotics is limited due to side effects and the possibility of antibiotic resistance. Hence, the identification of probiotics with excellent modulatory effects on the composition of the gut microbiome to inhibit TMA production might be useful for the prevention and treatment of AD.

Probiotics are defined as live microorganisms which, when administered or ingested in adequate amounts, confer a health benefit to the host. The contribution of intestinal microbiota to cognitive decline can be indirectly measured by the effects of probiotic supplementation on AD. Supplementation with probiotics ameliorated AD symptoms and improved biochemical markers in human patients [10] and animal models [11].

The mechanisms whereby TMAO contributes to pathological processes in AD have not been fully investigated. Therefore, in this study, we examined cognitive functions, TMAO synthesis, and the composition of the intestinal microflora in choline-treated mice. We also used the PROBIO database to predict the probiotics most likely to modify TMA/TMAO levels in plasma and behavioral symptoms. To test the effects of *Lactobacillus plantarum* (*L.* *plantarum*) on AD, we investigated the effects of *L.* *plantarum* and memantine in combination with *L.* *plantarum* on cognitive impairment, long-term potentiation (LTP), pathological deterioration, and TMAO synthesis in male transgenic AD mice. Lastly, we assessed the ability of *L.* *plantarum* alone or in combination with memantine to elicit the release of clusterin in plasma and relieve inflammation in the hippocampus.

## RESULTS

### Effects of choline supplementation on cognitive declines in C57BL/6J mice

**Figure 1:**
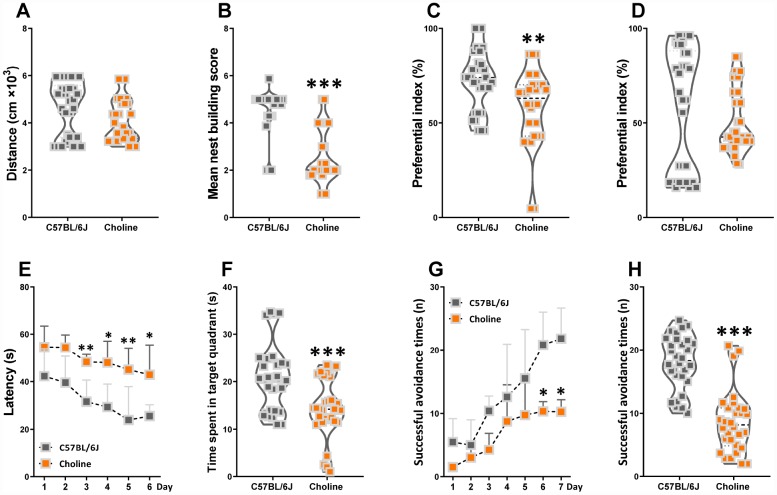
**Effects of choline supplementation on cognitive declines in C57BL/6J mice.** The spontaneous locomotor activity (**A**), nest building score (**B**), preferential index after training 1 hour (**C**) and 24 hours (**D**) in the phase of novel object test, latency in the learning phase (**E**) and time spent in target quadrant in the testing phase (**F**) of Morris water maze test, successful avoidance times in the learning (**G**) and testing phase (**H**) of shuttle box test. *P<0.05, **P<0.01, ***P<0.001, versus the C57BL/6J mice, by unpaired Student's *t*-tests. All values are mean ± S.D. n=30.

Choline supplementation ameliorates specific behavioral, neurological, and cognitive deficits [12]. However, higher choline intake is associated with poorer brain health and cognitive function among adults [13]. In our study here, the locomotor activity of C57BL/6J mice was the same as that of choline-treated mice (Figure 1A). In the nest building test, the mean score of the choline-treated group was lower than that of the C57BL/6J group (Figure 1B). To assess novel object recognition memory in these mice, we utilized the novel object recognition test (Figure 1C, 1D). Administration of choline caused a declined in short-term memory but had no effect on long-term object recognition. To examine spatial learning and memory in mice, we subjected them to the Morris water maze test (Figure 1E, 1F). In the training session, choline-treated mice exhibited a longer escape latency than C57BL/6J mice beginning on the third day. In the testing session, the swimming time within the target quadrant was lower for choline-treated mice than for C57BL/6J mice; however, there was no difference in the number of plate crossings, and swimming speed (data not shown) between the control group and choline-treated group. Finally, we subjected the mice to the shuttle-box test to evaluate their active avoidance (Figure 1G, 1H). Successful avoidance times were lower for the choline-treated group than for the control group beginning on the sixth day in the training phase as well as in the testing phase. These data indicated that learning and memory declined in C57BL/6J mice with long-term 1% choline administration.

### Effects of choline supplementation on TMAO synthesis in C57BL/6J mice

**Figure 2:**
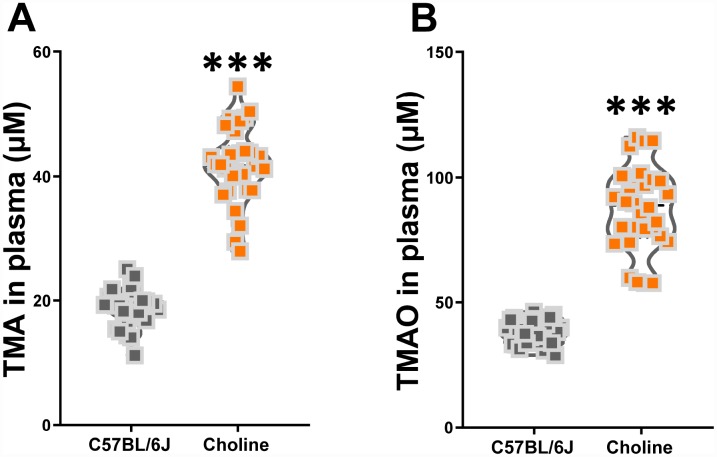
**Effects of choline supplementation on TMAO synthesis in C57BL/6J mice.** Plasma TMA (**A**) and TMAO (**B**) levels in C57BL/6J mice with or without choline supplementation (1%) as determined by LC/MS. ***P<0.001, versus the C57BL/6J mice, by unpaired Student's *t*-tests. All values are mean ± S.D. n=30.

To investigate the effect of choline supplementation on TMAO synthesis in C57BL/6J mice, we measured TMA and TMAO levels in plasma at three months after administration. Using liquid chromatography-tandem mass spectrometry (LC/MS), we found that plasma TMA and TMAO levels were much higher in choline-treated mice than those in the control group (Figure 2). Our results suggested that long-term choline supplementation increased TMAO synthesis levels in C57BL/6J mice.

### Correlation analysis of cognitive declines and reduced TMAO synthesis in choline-treated C57BL/6J mice

**Figure 3:**
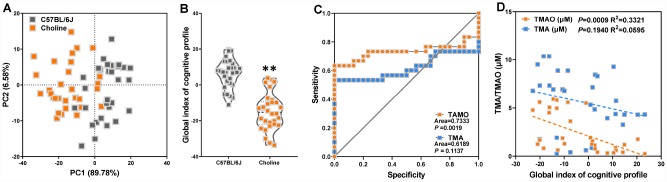
Increased plasma TMAO levels are associated with cognitive declines in C57BL/6J mice. Principal component analysis (PCA) data derived from behavioral experiments of mice with or without choline supplementation (**A**). Each axis was derived by PCA. Each point represents one of the mice with or without choline. Principal component 1 (variance explained: 89.78%), principal component 2 (variance explained: 6.58%) considered significant variance with a load below or equal to 0.50 (absolute value). The mean PCA scores of mice with or without choline (**B**). Receiver operating characteristic (ROC) curve plots for distinguishing choline-treated mice from control mice based on a TMA- or TMAO-based random forest model (**C**). Spearman correlation between the TMA, TMAO concentrations and cognitive impairments in WT and APP/PS1 mice (**D**). **P<0.01, versus the C57BL/6J mice, by unpaired Student's *t*-tests. All values are mean ± S.D. n=30.

To further assess the correlations between altered plasma TMA/TMAO and cognitive declines in choline-treated C57BL/6J mice, we first analyzed the experimental results of nest building test, novel object recognition test, Morris water maze test, and shuttle-box test using principal component analysis (PCA). PCA revealed principal components 1 (PC1, 89.78%) and 2 (PC2, 6.58%), which grouped mice into two distinct clusters (Figure 3A). The average of PCA scores across the aforementioned tests constitutes the global behavioral index of mice in our study. This index shows that the behavioral characteristics of choline-treated mice differed from those of C57BL/6J mice (Figure 3B). Subsequently, to test the discriminatory power of plasma TMA and TMAO levels in properly classifying mice groups, we calculated the average area under the ROC (Figure 3C). We noticed that the area under the curve (AUC) for each group was 0.6189 for TMA levels and 0.7333 for TMAO levels. AUC values <0.7 implies that the corresponding indicator is not a good metric to classify the study groups [14]. Spearman correlation analyses showed that between the circulating TMA, TMAO levels, and global index of cognitive profile in WT and APP/PS1 mice. TMAO levels were correlated with the global index of cognitive profile (Figure 3D). These results indicated that increased plasma TMAO levels are associated with cognitive declines in C57BL/6J mice.

### Effects of choline supplementation on microbial remodeling in C57BL/6J mice

**Figure 4:**
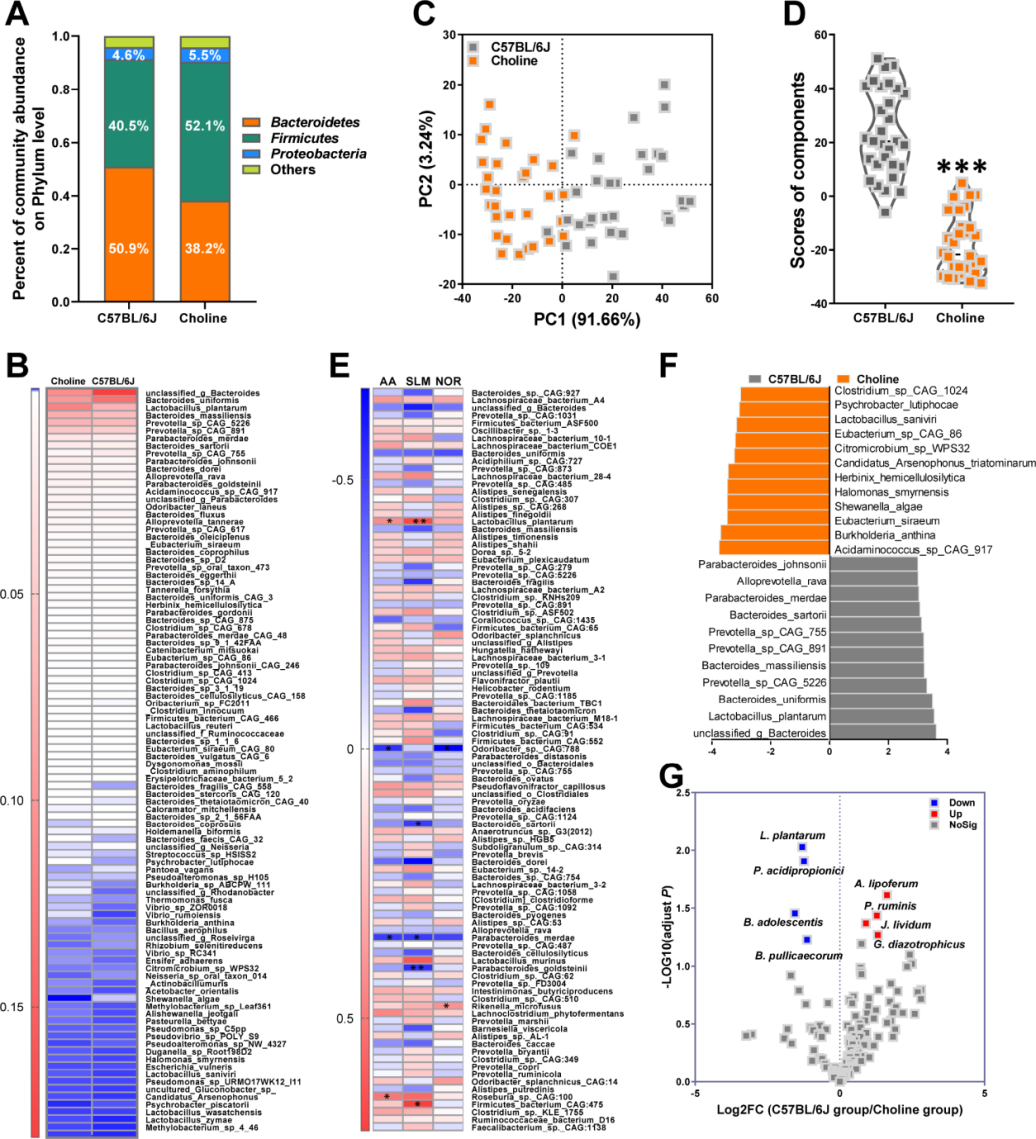
**Effects of choline supplementation on microbial remodeling in C57BL/6J mice.** Metagenomic analyses of feces at the phylum level (**A**). Heat map of metagenomic analyses of feces at the species level. The scale reveals the data as follows: red represents high values and blue represents low values for the percentages of reads that were classified at that rank (**B**). PCA at the species level in each group (**C**) and average PCA scores (**D**). ***P<0.001, versus the C57BL/6J mice, by unpaired Student's *t*-tests. All values are mean ± S.D. n=30. Correlation heat map demonstrating the association between the indicated microbiota taxonomic species and cognitive abilities from mice grouped by dietary status (chow and choline) (**E**). Red denotes a positive correlation, blue denotes a negative correlation, and white denotes no correlation. A single asterisk indicates a significant FDR-adjusted correlation at *P*≤0.05, and two asterisks indicate a significant FDR-adjusted correlation at *P*≤0.01. NOR=novel object recognition memory; SLM=spatial learning and memory; AA=active avoidance. The taxons most differentially abundant between the mice with or without choline at the species level were identified by linear discriminant analysis (LDA) coupled with effect size measurements (**F**). Choline-diet-enriched taxa are indicated with a negative LDA score (orange), and taxa enriched in the normal chow diet have a positive score (gray). Only taxa meeting an LDA significant threshold value of >3 are displayed. Volcano plots are used to visualize the differential probiotics expression between the mice with or without choline (**G**). The red points in the plot represent the differentially increased probiotics, the blue points represent the differentially decreased probiotics, the gray points represent the no changed probiotics with statistical significance.

The generation of circulating TMAO in plasma is dependent on intestinal microflora that can metabolize dietary choline into TMA, which is then converted into TMAO by members of the FMO family in the liver, among which FMO3 has been shown to be the most active enzyme [60]. Thus, to clarify the role of the intestinal microbiota in the choline-induced increase of TMAO levels, we investigated intestinal microbial populations in the gut at the phylum and species levels by metagenomic analyses. Analysis at the phylum level showed that the bacterial population of vehicle-treated C57BL/6J mice was dominated by *Bacteroidetes* (50.9%) and *Firmicutes* (40.5%), with a low level of *Proteobacteria* (4.6%) (Figure 4A). The relative amount of sequences assigned to *Firmicutes* was increased in metagenomes of choline-fed C57BL/6J mice at the expense of *Bacteroidetes* (Figure 4A). Species-level analysis revealed that choline induced a decrease in the relative abundances of *Clostridium_sp_CAG_1024*, *Psychrobacter_lutiphocae, Lactobacillus_saniviri, Eubacterium_sp_CAG_86, Citromicrobium_sp_WPS32, Candidatus_Arsenophonus_triatominarum, Herbinix_ hemicellulosilytica, Halomonas_smyrnensis, Shewanella_ algae, Eubacterium_siraeum, Burkholderia_anthina, Acidaminococcus_sp_CAG_917* (Figure 4B and 4F). Choline administration resulted in an increase in the relative abundances of *Parabacteroides_ johnsonii, Alloprevotella_rava, Parabacteroides_ merdae, Bacteroides_sartorii, Prevotella_sp_CAG_ 755, Prevotella_sp_CAG_891, Bacteroides_massiliensis, Prevotella_sp_CAG_5226, Bacteroides_uniformis, Lactobacillus_plantarum, unclassified_g_Bacteroides* (Figure 4B and 4F). We used PCA to determine the influences of choline on intestinal microbial populations in the gut at the species levels. The PC1 and PC2 grouped mice from the different groups into two distinct clusters (Figure 4C). The overall gut microbiota of choline-fed C57BL/6J mice, standardized for their performance, is presented in Figure 4D. The index clearly shows that the intestinal microbial characteristics of choline-treated mice differed from that of C57BL/6J mice. Spearman correlation analyses revealed that, at the species level, there were eight bacterial species correlated with cognitive abilities, including one negatively correlated (*Odoribacter_sp_CAG_788*) and one positively correlated (*Rikenella_microfusus*) with object recognition memory, three negatively correlated (Bacteroides_sartorii, *Parabacteroides_merdae* and *Parabacteroides_ goldsteinii*) and two positively correlated (*Lactobacillus_plantarum* and *Firmicutes_bacterium_ CAG_475*) with spatial learning and memory, and two negatively correlated (*Odoribacter_sp_CAG_788* and *Parabacteroides_merdae*) and two positively correlated (*Lactobacillus_plantarum* and *Alistipes_putredinis*) with the active avoidance response (Figure 4E). Of these, *L.* *plantarum* was positively correlated with cognitive performance in the Morris water maze and shuttle-box tests.

Medicinal foods, specifically probiotics, have recently materialized as a tool to manage various intestinal disorders. Indeed, the synthesis of microflora-dependent TMAO from choline-containing precursors might be reduced by some probiotics [15]. A probiotics database termed PROBIO is a knowledge-based database of probiotics functions and lineages [16]. In this study, clusters of probiotics were annotated for their characteristic sequences by best-hit against a PROBIO database and identified and grouped into 135 probiotics (Figure 4G). For choline-fed mice, the relative abundances of *Lactobacillus_plantarum, Propionibacterium acidipropionici, Butyricicoccus pullicaecorum,* and *Bifidobacterium adolescentis* were lower, and the relative abundances of *Anthinobacterium lividum, Pseudobutyrivibrio ruminis, Gluconacetobacter diazotrophicus,* and *Azospirillum lipoferum* were higher than those in the control group. Notably, *L.* *plantarum* was associated with cognitive declines and plasma TMAO levels in choline-treated mice (Supplementary Figure 1). Thus, in subsequent experiments, we attempted to assess the effects of *L.* *plantarum* on cognitive impairments in an AD mouse model via modulation of TMAO generation.

### Effect of *L. plantarum* supplementation on cognitive impairments in APP/PS1 mice

**Figure 5:**
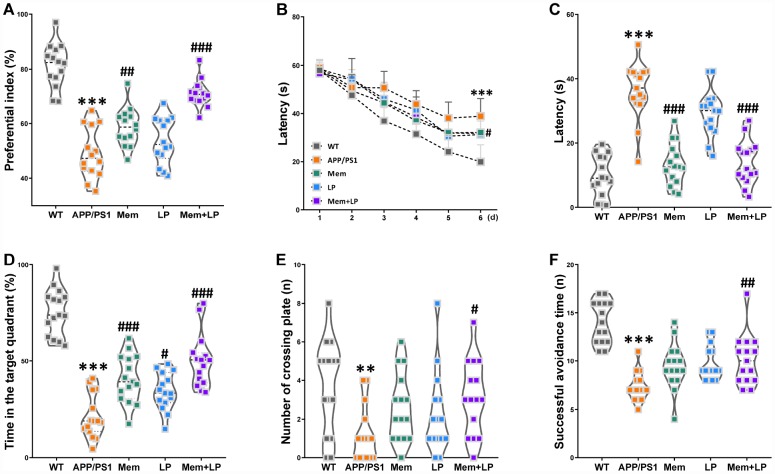
**Effect of *L. plantarum* supplementation on cognitive impairments in APP/PS1 mice.** APP/PS1 mice were treated with memantine, *L. plantarum*, or memantine+ *L. plantarum*. The preferential index after training 1 hour (**A**) in the phase of novel object test, latency in the learning phase (**B**), latency (**C**), time spent in target quadrant (**D**) and number of crossing plate (**E**) in the testing phase of Morris water maze test, successful avoidance times in testing phase (**F**) of shuttle box test. **P<0.01, ***P<0.001, versus the WT mice, by unpaired Student's *t*-tests. ^#^*P*<0.05, ^##^*P*<0.01, ^###^*P*<0.001, versus the APP/PS1 mice, by one-way ANOVA analysis followed by Dunnett’s *post hoc* test or a two-way repeated-measures ANOVA with *post-hoc* Tukey multiple comparisons test. All values are mean ± S.D. n=15. WT=wild type; Mem=memantine; LP=*L. plantarum*.

To determine the effect of *L. plantarum* treatment on cognitive behavior of a transgenic AD mouse model, we subjected mice to the novel object recognition test, the Morris water maze test, and the shuttle-box test. Six-month-old male WT non-transgenic littermates and APP/PS1 mice were selected, which received sterilized PBS, a dose of 1 mg/mL memantine, 1×10^9^ CFU/mL *L.* *plantarum*, and 1 mg/mL memantine and 1×10^9^ CFU/mL *L. plantarum*, respectively. Treatments were administrated to the mice once a day by oral gavage. After a three-month treatment, novel object recognition testing revealed a decreased preferential index among APP/PS1 mice in short-term memory (Figure 5A). However, treatment with memantine alone or in combination with *L. plantarum* improved these animals’ short-term memory. In the Morris water maze test, APP/PS1 mice had longer escape latencies compared with WT mice on the last learning day, and the latencies in the memantine-, *L. plantarum*-, or combination-treated APP/PS1 mice were shorter than those in the model group (Figure 5B). In the testing phase, the mean escape latency was longer (Figure 5C), the time in the target quadrant was shorter (Figure 5D), and the number of plate crossings diminished (Figure 5E); however, there was no difference in swimming speed (data not shown) in APP/PS1 mice compared with WT mice. Escape latencies were reduced in combination- or memantine-treated APP/PS1 mice, and the time in the target quadrant was reversed in mice treated with memantine, *L. plantarum*, or combination while the number of plate crossings expanded only in the combination-treated group. In the shuttle-box test, there was a decrease in avoidance times in APP/PS1 compared with WT mice; however, this was rescued after the administration of combination treatments (Figure 5F). Spearman correlation analysis showed that between the circulating TMA/TMAO/FMAO3 levels and global index of cognitive profile in WT and APP/PS1 mice. TMA and TMAO levels were correlated with the global index of cognitive profile (Supplementary Figure 2). These findings showed that *L. plantarum* augmented the effects of memantine in the treatment of short-term object recognition memory, spatial learning and memory, and active avoidance response deterioration in AD mice.

### Effect of *L. plantarum* supplementation on long-term potentiation (LTP) decline in APP/PS1 mice

**Figure 6:**
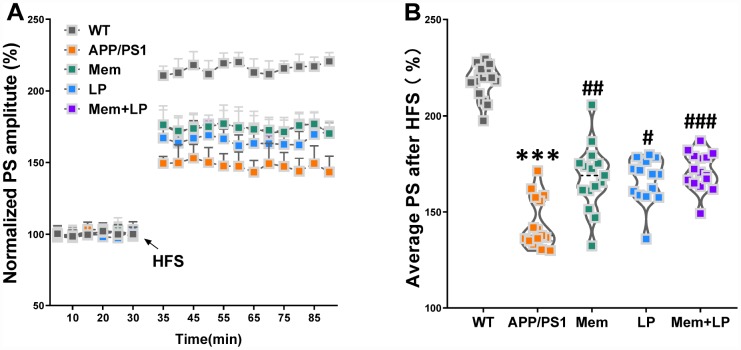
**Effect of *L. plantarum* supplementation on long-term potentiation (LTP) decline in APP/PS1 mice.** The population spike (PS) amplitudes and LTP induction were recorded in WT and APP/PS1 mice (**A**). Summary of average PS amplitude (31-90 minutes) from all experiments shown in A (**B**). ***P<0.001, versus the WT mice, by unpaired Student's *t*-tests. ^#^*P*<0.05, ^##^*P*<0.01, ^###^*P*<0.001, versus the APP/PS1 mice, by one-way ANOVA analysis followed by Dunnett’s *post hoc* test. All values are mean ± S.D. n=15. W=wild type; Mem=memantine; LP=*L. plantarum*.

LTP is a putative neuromechanism of associative memory formation and preservation related to cognition capacity [17]. The average population spike (PS) amplitude of APP/PS1 mice decreased after HFS (Figure 6). On the other hand, the average PS amplitude of APP/PS1 mice was elevated by the treatment with memantine, *L. plantarum*, and combination, compared to that of controls. Spearman correlation analysis showed that TMA and TMAO levels correlated with LTP (Supplementary Figure 3).

### Effects of L. plantarum supplementation on hippocampal Αβ plaques in APP/PS1 mice

**Figure 7:**
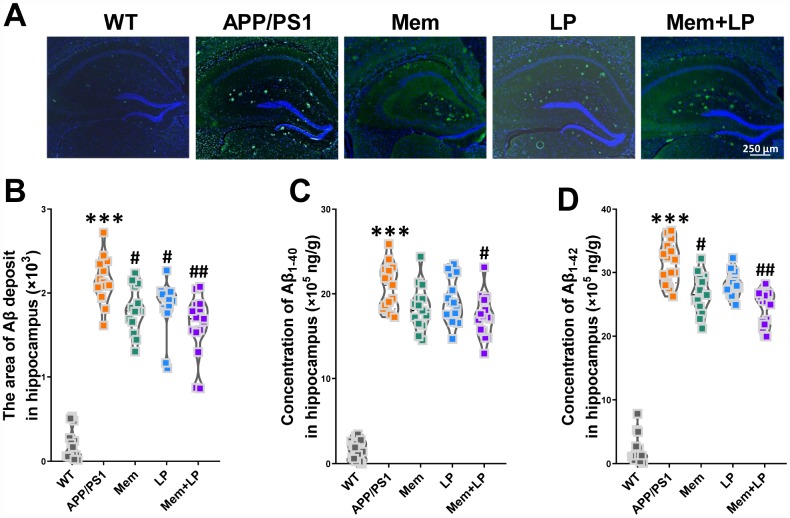
**Effect of *L. plantarum* supplementation on Αβ deposition in APP/PS1 mice.** Representative immunofluorescence staining images showing Αβ plaques (green and indicated by red arrows) in the hippocampus of WT and APP/PS1 mice (**A**). Quantitative analysis of Αβ plaques (**B**), Aβ_1-40_ (**C**) and Aβ_1-42_ (**D**) in the hippocampus was used by Image Pro Plus 6.0 software and AlphaLISA assay. ***P<0.001, versus the WT mice, by unpaired Student's *t*-tests. ^#^*P*<0.05, ^##^*P*<0.01, versus the APP/PS1 mice, by one-way ANOVA analysis followed by Dunnett’s post hoc test. All values are mean ± S.D. n=15. WT=wild type; Mem=memantine; LP=*L. plantarum*.

Αβ plaques in the cerebrum is the most common pathological hallmark of AD in patients and animal models. We performed immunofluorescent staining of brain slices stably expressing human Aβ with the antibody 6E10, which labels Aβ. Our results demonstrated that APP/PS1 mice developed numerous Αβ plaques in the brain at nine months while WT mice did not (Figure 7A). Memantine-, *L. plantarum*-, and combination-treated APP/PS1 mice showed a reduction of Αβ plaques in the hippocampus (Figure 7B). AlphaLISA assay showed that the concentration of Aβ_1-42_ and Aβ_1-40_ in the hippocampus of APP/PS1 mice was higher than that in WT mice (Figure 7C and 7D). Spearman correlation showed that TMA and TMAO levels correlated with Αβ plaques, Αβ_1-42_ and Αβ_1-40_ levels in the hippocampus (Supplementary Figure 4). These data indicated that while all treatments inhibited Αβ, combination treatment showed the strongest effect and Memantine alone showing the weakest.

### Effects of *L. plantarum* supplementation on hippocampal neurons and plasticity in APP/PS1 mice

**Figure 8:**
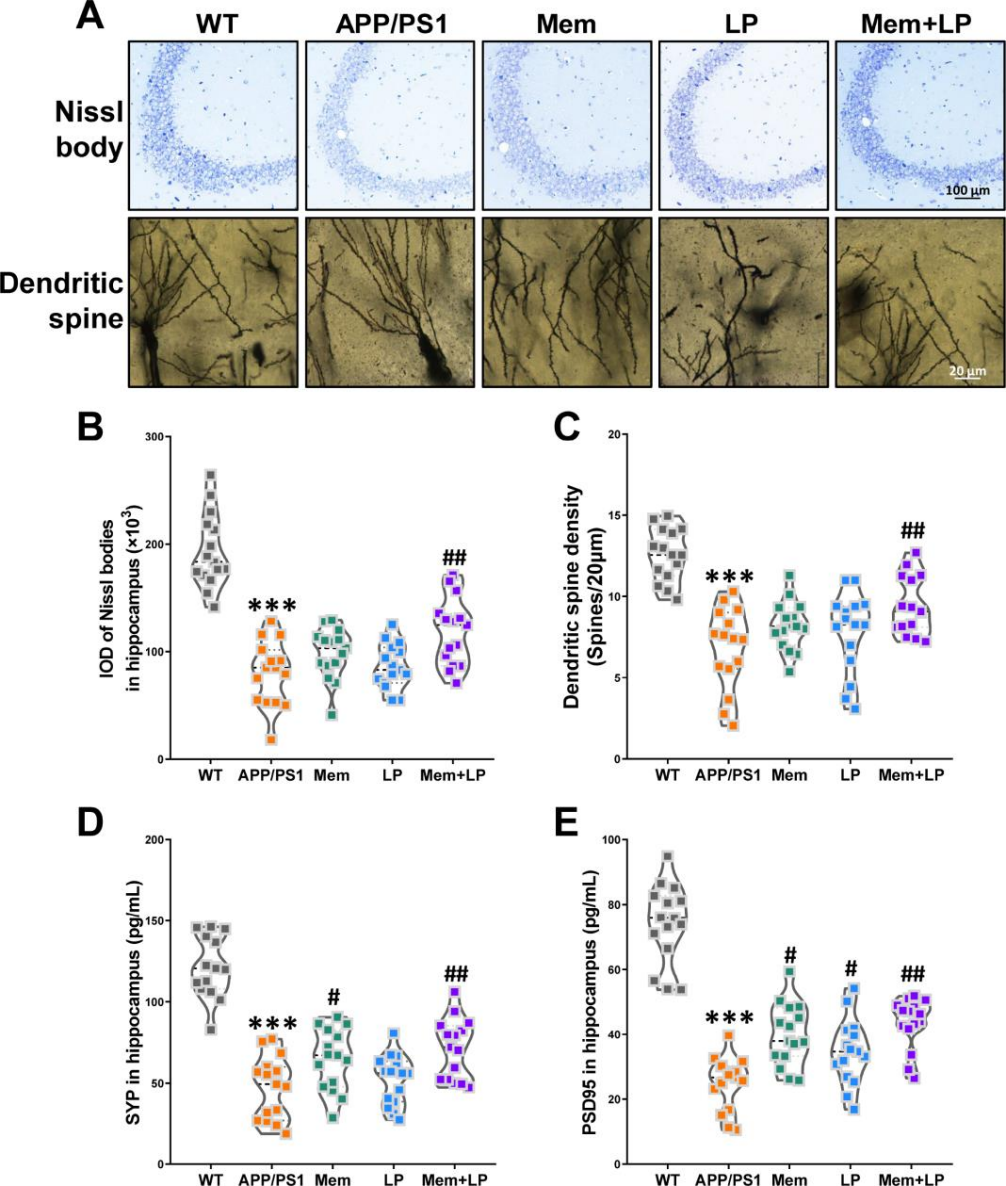
**Effects of *L.plantarum* supplementation on hippocampal neuron and plasticity in APP/PS1 mice.** Representative images of Nissl staining images (upper panel) and dendritic spines (lower panel) under microscopy in the hippocampus of WT and APP/PS1 mice (**A**). Quantification of and Nissl bodies (**B**) and mean dendritic spine density (**C**). The expression of hippocampal synaptophysin (**D**) and PSD95 protein (**E**). ***P<0.001, versus the WT mice, by unpaired Student's *t*-tests. ^#^*P*<0.05, ^##^*P*<0.01, versus the APP/PS1 mice, by one-way ANOVA analysis followed by Dunnett’s *post hoc* test. All values are mean ± S.D. n=15. WT=wild type; Mem=memantine; LP=*L. plantarum*.

To determine the effects of *L. plantarum* on hippocampal neurons and plasticity, we measured the amount of Nissl bodies and dendritic spine density using Nissl and Golgi staining. Nissl staining showed neuropathological alterations in the hippocampus of APP/PS1 mice compared to WT mice (Figure 8A), such as fewer Nissl bodies (Figure 8B), which was partially rescued by combination treatment. Golgi staining demonstrated that APP/PS1 mice had decreased dendritic spine density in the hippocampus, which was attenuated by combination treatment (Figure 8C). We also investigated the levels of synaptic proteins, including synaptophysin and PSD95 (Figure 8D and 8E). Spearman correlation showed that TMA and TMAO levels correlated with hippocampal neuron integrity and plasticity (Supplementary Figure 5). We found that APP/PS1 mice had decreased levels of synaptophysin and PSD95, which were improved by memantine and combination treatments. On the other hand, *L. plantarum* administration elicited only a modest increase in PSD95 expression. These data revealed that addition of *L. plantarum* to the treatment of APP/PS1 mice treated with memantine protected hippocampal neurons and plasticity.

### Effects of *L. plantarum* supplementation on TMAO synthesis in APP/PS1 mice

**Figure 9:**
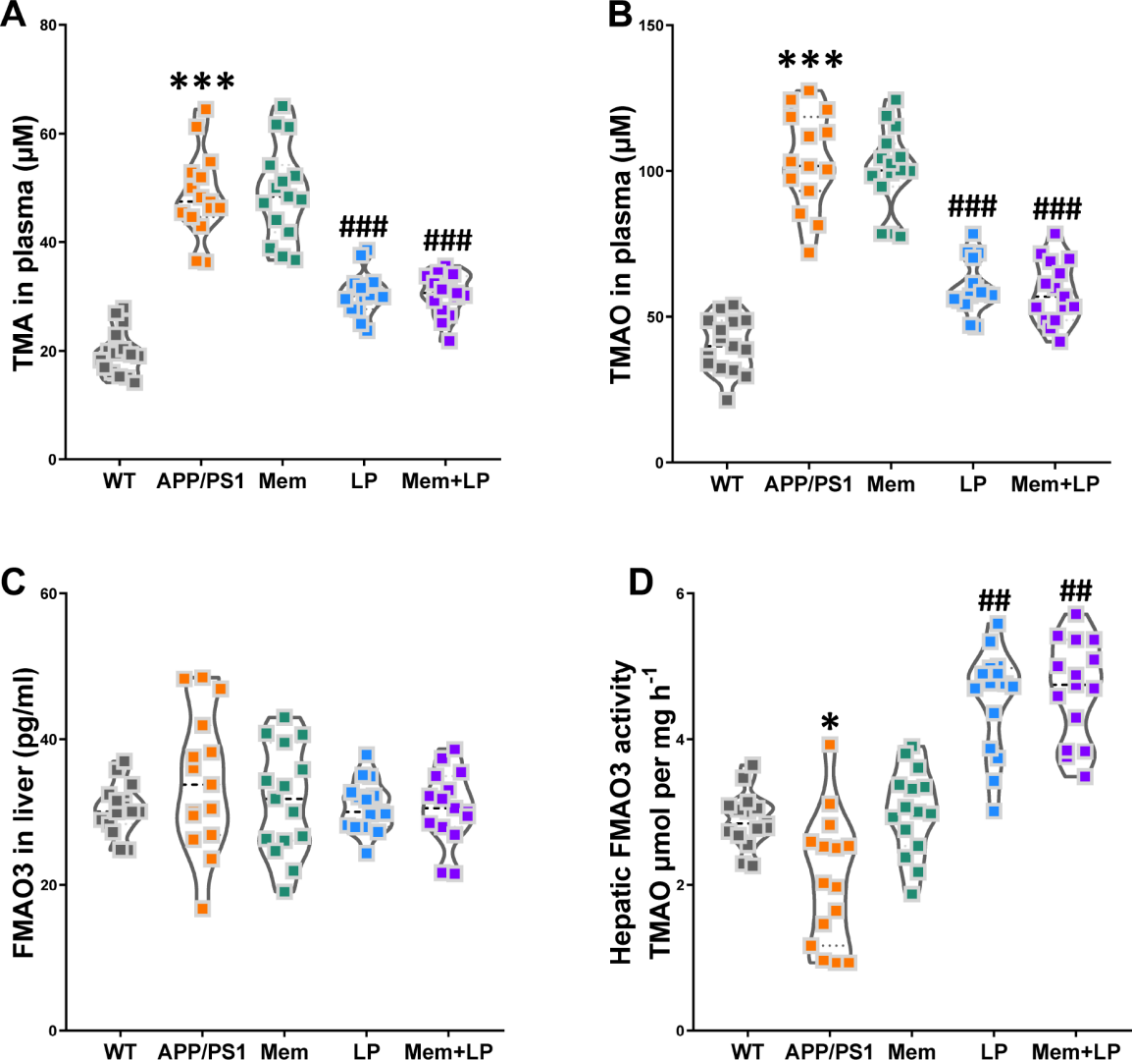
**Effects of *L. plantarum* supplementation on TMAO synthesis in APP/PS1 mice.** The concentration of TMA (**A**), TMAO (**B**) in plasma, and FMO3 (**C**) in the liver. Liver FMO activity, assessed as described in Materials and Methods (**D**). ***P<0.001, versus the WT mice, by unpaired Student's *t*-tests. ^#^*P*<0.05, ^##^*P*<0.01, ^###^*P*<0.001, versus the APP/PS1 mice, by one-way ANOVA analysis followed by Dunnett’s *post hoc* test. All values are mean ± S.D. n=15. WT=wild type; Mem=memantine; LP=*L. plantarum*.

Alterations in the composition of gut flora can reduce intestinal TMA levels, leading to decreased TMAO synthesis in the liver. As shown in Figure 9, treatment with *L. plantarum* or a combination of memantine and *L. plantarum* markedly decreased TMA and TMAO levels and increased hepatic FMO activity while FMO3 levels in the liver remained unchanged. These results were consistent with our observation in C57BL/6J mice, suggesting an important role for the intestinal microbiome in reducing TMAO levels.

### Effects of *L. plantarum* supplementation on the levels of clusterin in plasma and inflammatory status in the hippocampus of WT and APP/PS1 mice

**Figure 10:**
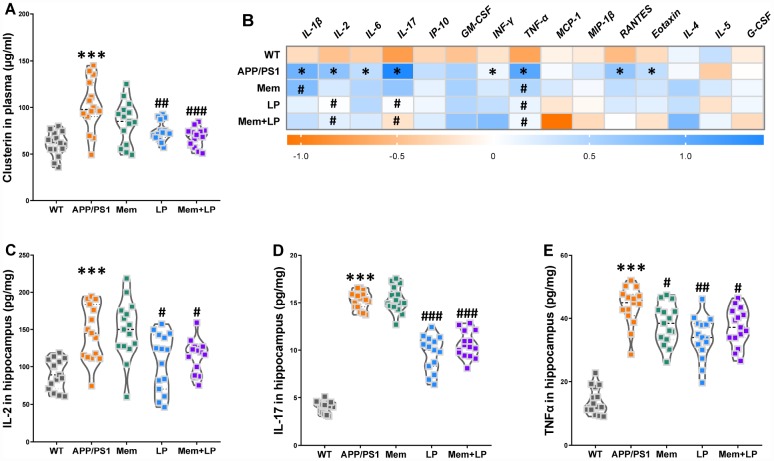
**Effects of *L. plantarum* supplementation on the levels of clusterin in plasma and inflammatory status in the hippocampus of WT and APP/PS1 mice.** The concentration of plasma clusterin (**A**). Heat map of cytokine concentrations (*z*-score) in the hippocampus (**B**). * represents a difference between WT and APP/PS1 mice, # represents a difference between APP/PS1 mice and treated APP/PS. The concentration of interleukin (IL)-2 (**C**), IL-17 (**D**) and tumor necrosis factor α (TNFα) (**E**) in the hippocampus. ***P<0.001, versus the WT mice, by unpaired Student's *t*-tests. ^#^*P*<0.05, ^##^*P*<0.01, ^###^*P*<0.001, versus the APP/PS1 mice, by one-way ANOVA analysis followed by Dunnett’s *post hoc* test. All values are mean ± S.D. n=15. WT=wild type; Mem=memantine; LP=*L. plantarum*. IL=interleukin; IP=interferon-induced protein; GM-CSF=granulocyte-macrophage colony-stimulating factor; IFNγ=interferon-γ; TNF-α=tumor necrosis factor α; MCP-1=monocyte chemotactic protein-1; RANTES=regulated upon activation normal T cell expressed and secreted factor; MIP-1β=macrophage inflammatory protein-1β; G-CSF=granulocyte colony-stimulating factor.

Increased clusterin levels in plasma promoted the accumulation of fibrillar Aβ aggregates in AD mouse brains while clopidogrel inhibited it [18]. In our study, APP/PS1 mice had a higher concentration of clusterin in plasma than WT mice (Figure 10A), which was reduced by treatment with *L. plantarum* or the combination of memantine and *L. plantarum*. Given established correlations between clusterin levels in plasma and numerous immunomodulatory regulators, clusterin might constitute a potential therapeutic target to treat irregular immune responses in the AD cerebrum [19]. Therefore, we used multiplex bead analysis to comprehensively measure inflammation in the hippocampus (Figure 10B). We found a significant increase in hippocampus levels of proinflammatory cytokines, including the classical mediators IL-1β, IL-2, IL-6, IL-17, IFNγ, TNF-α, RANTES, and Eotaxin in APP/PS1 mice. On the other hand, neuroinflammation in the hippocampus was reduced in *L. plantarum-* or combination-treated mice, as shown by the reduction in IL-2 (Figure 10C), IL-17 (Figure 10D), and TNF-α (Figure 10F) concentrations. Thus, this suggests that *L. plantarum* attenuated neuroinflammation by reducing clusterin levels via inhibition of TMA production and TMAO synthesis.

## DISCUSSION

In the current study, we found that *L. plantarum* decreased TMAO levels by suppressing gut microbial TMA secretion via remodeling of gut microbiota in transgenic AD mice, thereby attenuating cognitive impairments and pathological deterioration. To the best of our knowledge, this is the first study demonstrating the role of TMAO in *L. plantarum*-induced protection against AD. Moreover, the addition of *L. plantarum* to memantine treatment potentiated the therapeutic benefits of memantine alone by decreasing the concentration of Αβ_1-42_ and Αβ_1-40_, protecting hippocampal neurons and plasticity, inhibiting TMAO synthesis, and alleviating neuroinflammation.

Dietary choline is metabolized by the gut microbiota into TMA, which is converted by FMO3 into hepatic TMAO. The intestinal metabolite TMAO contributes to the pathogenesis of AD [20]. Indeed, CFS TMAO levels are higher in AD patients than in cognitively-unaffected individuals [6] and correlated with AD susceptibility and pathogenicity [5]. Furthermore, elevated circulating TMAO reduced hippocampal antioxidant enzyme levels [21], induced cognitive dysfunction, and accelerated brain aging [22]. Here, feeding C57BL/6J mice diets enriched in choline resulted in declines in novel object recognition memory, spatial learning and memory, and active avoidance response. In addition, dietary choline supplementation elevated TMA and TMAO levels in plasma but cognitive decline correlated only with the latter.

The gut microbiota and its host are symbiotically related. The intestine has been defined as the second brain, because it can affect psychological, emotional, and cognitive homeostasis [23–25], evidencing the functional influence the gut and the cerebrum can have on each other. Alterations in the relative abundance of intestinal flora correlated with increased concentrations of phosphorylated *tau* and with an increased Aβ_1-40_/Aβ_1-42_ ratio in AD patients [26]. Similarly, intestinal mucosal barrier dysfunction correlated with increased fecal calprotectin concentrations [27], and a specific gut microbiome composition correlated to AD histological and behavioral hallmarks in APP/PS1 mice [28]. Moreover, colonization of germ-free APP transgenic mice with intestinal flora from conventionally-raised APP transgenic mice increased cerebral Aβ pathology, and vice versa [29]. Hence, gut microbiota alternations might accelerate the appearance of AD-like cognition decline and lesions. Our study showed that there were that gut microbiota were differentially abundant in control mice and choline-treated mice. Furthermore, we found that abundance of *L.* *plantarum* correlated with cognitive declines and plasma TMAO levels in choline-administrated mice based on data from the PROBIO database.

Probiotics are regarded as a novel and harmless way of stabilizing a healthy gut microbiota by competing with other intestinal bacteria for binding sites or receptors on the mucosa [30], or by secreting metabolic compounds that restrain the growth of other microorganisms [31, 32]. Probiotic administration can also induce neuronal plasticity and elicit improvements in behaviors associated with psychiatric conditions. For instance, probiotics appear to protect neurogenesis in the brain as measured by increases in brain-derived neurotrophic factors, which in turn can reduce inflammatory cytokines and oxidative stress [33]. In addition to improving the immune functions of the host, probiotics can balance gut microbiota to produce states that are advantageous to beneficial microorganisms [34], as seen in clinical studies. Indeed, probiotic supplementation improved cognitive and emotional symptoms in AD patients as well as metabolic function, supporting the hypothesis that probiotics can activate specific brain regions involved in the control of emotion and sensation [35]. Some studies have shown that western diets can promote anxiety but supplementation with *L. helveticus* can prevent this effect [36]. Similarly, supplementation with various probiotic strains has been observed to improve cognitive functions and alleviate symptoms in several diseases such as diabetes [37], among others [38]. VSL#3, a probiotic mixture containing eight Gram-positive probiotic strains, modulated the expression of genes that impact inflammatory and neuronal plasticity processes in brain tissue, attenuated age-related deficits in LTP, and decreased microglial activation markers while increasing synapsin expression [39]. Here, we observed that *L. plantarum* augmented the effects of memantine in the treatment of cognitive deterioration in APP/PS1 mice. Moreover, combination treatment with memantine and *L. plantarum* attenuated hippocampal LTP deficits, decreased intracerebral Aβ deposits and the abundance of other species including oligomers, and reduced Αβ_1-42_ and Αβ_1-40_ levels while protecting neuronal integrity and plasticity in the hippocampus. Therefore, our findings suggest that *L. plantarum* might augment the therapeutic effects of memantine on AD-like cognition and neuropathology impairments in APP/PS1 mice.

Emerging evidence has revealed that gut microbiota, through the production of TMAO, directly contribute to platelet hyperreactivity [7]. Moreover, circulating TMAO was not strictly correlated with differences between vegetarian and omnivorous diets [8, 40]. Yet, TMAO levels might be affected by modifications in gut microbiota composition, which possibly modulate TMA production [41]. TMAO increases the risk of thrombosis [7], disruption of hormonal homeostasis [42], and inflammation [43]. Here, we found that *L. plantarum* and the combination of memantine and *L. plantarum* inhibited TMAO synthesis by decreasing gut microbial TMA production through gut microbiota modulation, which subsequently attenuated cognition and neuropathology deficits in APP/PS1 mice. Meanwhile, a recent report revealed that 3,3-dimethyl-1-butanol could reduce TMAO levels by inhibiting the formation of TMA from microbes in aged rodents, thereby attenuating inflammation and oxidative stress [44]. These results suggested that targeting the intestine microbial generation of TMA distinctively through probiotic supplementation may serve as an effective therapeutic strategy for AD.

Clusterin is a versatile glycoprotein that promotes AD progression and modifies the structure of Αβ [45], consistent with results from clinical trials [46, 47]. The effects of clusterin on Aβ accumulation and might depend on the ratio between their levels [48, 49]. Similarly, fibrillar Aβ deposits in PDAPP mice expressing clusterin were more abundant than clusterin-knockout PDAPP mice, even though the general abundance of deposits was comparable, suggesting that clustering might stabilize a specific subset of Aβ conformations [50]. Clusterin knockout in APP/PS1 mice demonstrated a noticeable decrease in cerebral Aβ deposits and decreased inflammation [51]. Regarding immune mechanisms, complement components were identified as particular risk factors for AD by GWAS [52, 53], with clusterin modulating the complement system and various cytokines, including TNF-α [54], IL-2 [55], IL-17 [56], and membrane attack complex [57]. In our study here, we found high levels of clusterin and proinflammatory cytokines in the hippocampus of APP/PS1 mice and observed that treatment with *L. plantarum* or a combination of memantine and *L. plantarum* reduced such levels.

A limitation of our study is that we do not address the molecular mechanisms underlying the therapeutic effects we have uncovered *L. plantarum* and combination treatments. We speculate that TMAO might indirectly alter signaling pathways by altering the conformation of its protein partners. Indeed, TMAO can directly stabilize protein folding and induce conformational changes in proteins by functioning as a small-molecule protein chaperone [58, 59]. It is thus conceivable that TMAO might modify numerous signaling pathways without directly activating a specific TMAO receptor. Nonetheless, our results here showing that *L. plantarum* augments the beneficial effects of memantine on AD open a new avenue of research and highlight the intestinal microbiota as an exciting target for pharmacological or dietary interventions to decrease the risk of AD.

## MATERIALS AND METHODS

### Animals and treatments

Eight-week-old male C57BL/6J mice (*n*=60) were obtained from Jackson Laboratory (Bar Harbor, USA). For all the experiments, mice were kept and fed individually and had random access to potable water. Mice in the control group (*n*=30) were fed a standard chow diet, and the model group (n=30) was fed a chow diet supplemented with 1% choline for three months. Mice were weighed every week and were maintained at the animal experimental center of Harbin medical university in a temperature-controlled environment (22 ± 2°C), with a 12-h light/dark cycle. Animal experiments were carried out in accordance with the references in the Guide for the Care and Use of Laboratory Animals published by the National Institutes of Health and were approved by the Institutional Animal Care and Use Committee of Harbin medical university.

Six-month-old male wild type (WT) non-transgenic littermates and PrP-hAβPPswe/PS1^ΔE9^ transgenic (APP/PS1) mice with a C57BL/6 genetic background were obtained from Jackson Laboratory (Bar Harbor, USA). *Lactobacillus plantarum* ATCC 8014 was purchased from ATCC, via the Beijing BioChen Co. Ltd. *L.* *plantarum* was stored in 15% glycerol at -80 °C and anaerobically cultured in DeMan-Rogosa-Sharpe (MRS) agar at -37 °C. *L.* *plantarum* cell pellets were collected and washed with PBS (8000 rpm for 15 min). The harvested pellet was resuspended in PBS comprising 15% glycerol to prepare the concentration of 5×10^9^ CFU/mL and subsequently stored at -80 °C until use for oral administration to mice. After a three-day adaptation period, the mice were randomly separated into seven groups (*n*=15 mice per group) as follows: control group (WT mice) and model group (APP/PS1 mice), which received sterilized PBS (pH 7.4); memantine group (APP/PS1 mice), which received a daily dose of 1 mg/mL memantine (Sigma-Aldrich, USA); *L.* *plantarum* group (APP/PS1 mice), which received a daily dose of 1×10^9^ CFU/mL *L.* *plantarum*; combination group (APP/PS1 mice), which received a daily dose of 1 mg/mL memantine and 1×10^9^ CFU/mL *L. plantarum*. Treatments were administrated once a day for 12 weeks by oral gavage. Following behavioral tests and electrophysiological measurements, mice were placed in a sealed chamber and euthanized via isoflurane inhalation and cervical dislocation. The hemisphere and plasma of each mouse were collected for biochemical and histochemical analyses.

### Behavioral experiments

### Spontaneous locomotor activity test

The spontaneous locomotor activity proceeded for 20 min. Spontaneous locomotor behavior was recorded by a video-based behavior monitoring system (XinRan Technology, China).

### Nest building test

Mice were placed into individual testing chambers with one nestled (4 cm squares). The nesting score was calculated at the following morning by a pre-determined measuring scale (least: 1, best: 6).

### Novel object recognition test

The testing paradigm of the object recognition test consisted of three phases: familiarization, training, and testing. Generally, to be familiar with the testing condition, mice freely explored an empty chamber (10 min daily) for two days. On the third day, mice explore two similar objects (object A1 and A2) that are averagely placed at opposing ends of the chamber. Object exploration was described as the time mice spent bodily contacting the object within 0.2 cm. Each mouse explored similar objects for 10 min. After a 1-hour and 24-hour training-to-testing interval, a novel object (object B or C) was taken the place of one of the same objects. The preferential index (time on object B or C/(time on object B or C＋time on object A)×100%) was calculated to evaluate object recognition memory in 5 min phase.

### Morris-water maze test

Morris-water maze test was conducted in a white circular pool (diameter: 90 cm and depth: 45 cm). The pool was filled with 30-cm-depth water. The water temperature was kept at 20 ± 1°C by adding ice water. The platform of diameter 6 cm was placed 1 cm underneath the water. The training and testing periods were 60 s. In the training period, mice finished four trials daily for five consecutive days with a hidden platform. Mice that did not discover the platform within 60 s were guided towards the hidden platform and put onto the platform for 5 s. In the testing period, the platform was removed and the mice were allowed to search for the disappeared platform for 60 s. The latency to find the hidden platform in the training and testing periods and the time spent in the target quadrant was recorded and analyzed.

### Shuttle-box test

In the shuttle box test, all avoidance training phases consisted of 30 trials with the following parameters: 15 s tone (50 dB) and light (9W), 5 s foot shock (0.25 mA), and 15 s training-to-testing interval. A testing phase with the same parameters without foot-shock, after the 7 consecutive days, was carried out. The argument automatically recorded was the successful active avoidance times.

### Electrophysiology

The mice were anesthetized by 1.0 g/kg urethane intraperitoneal injection and positioned in a stereotaxic frame (Narishige). A bipolar stimulating electrode was inserted in the perforant path (anterior to lambda: 0.6 mm, to midline: 2.4 mm, to brain surface: 1.7-2.1 mm). The evoked potentials were received with an electrode at the dentate gyrus (-2.0 mm, 1.0 mm, 1.7-2.2 mm). The electrical stimulus produced by the stimulator. The pulses (0.1 ms, 1/60 Hz) transmitted through an isolator (Nihon Kohden) to provide a steady electric current. The evoked responses were amplified and low-pass filtered (1000 Hz, Axon Instruments), then transmitted through a data acquisition system (Digidata 1200, Axon Instruments). After obtaining a steady stimulus-response curve, a 1/2 maximum population spike (PS) was applied. Following a recording (30 min), the long-term potentiation (LTP) was induced by high-frequency stimulation (HFS), and the PSs of each mouse were recorded (31-90 minutes).

### Histochemical and biochemical analyses

### Immunofluorescence

Hemispheres were collected and fixed in 4% paraformaldehyde, then embedded by paraffin. The 5-μm-thick tissue sections were prepared. Subsequently, sections were incubated overnight at 4 °C with primary antibodies for β-amyloid (6E10, Biolegend, USA). After being washed three times in PBS, the sections were incubated with goat anti-mouse IgG HRP (1: 1000, ZSGB-Bio, Beijing, China) for 2 h at room temperature, then incubated with Opal^TM^ 6-Color Fluorescent IHC Kit (PerkinElmer, USA) for 15 min, and mounted with DAPI-containing medium. Sections were digitized by a fluorescence imaging microscopy (Vectra 2, PE-Caliper LS, USA). The area of Aβ plaque was calculated by Image Pro Plus 6.0 software.

### Nissl staining

The sections were stained by 0.5% cresyl violet acetate (Beyotime, China) and photographed using a transmission electron microscope (TEM)(Hitachi, Japan). The integrated optical density (IOD) of Nissl bodies in the hippocampus was calculated using Image Pro Plus 6.0 software.

### Golgi staining

To investigate dendritic spine density, the cerebrums were transferred to the Golgi staining solution. Golgi staining was performed in accordance with the instructions for the FD Rapid Golgistain™ Kits (FD NeuroTechnologies, USA) on unperfused cerebrum tissue.

### Soluble Aβ analysis

The Aβ AlphaLISA assay was performed in this study. The concentration of Aβ_1-40_ and Aβ_1-42_ in the hippocampus were quantified using the Aβ_1-40_ (AL275C, PerkinElmer, USA) and Aβ_1-42_ (AL276C, PerkinElmer, USA) kits according to the manufacturer instructions.

### Enzyme-linked immunosorbent assay

The concentration of FMO3 in the liver was assessed using a mouse flavin containing monooxygenase 3 ELISA kit (MBS9327471, MyBioSource, USA). The levels of clusterin in plasma were assessed with a mouse clusterin quantikine ELISA kit (MCLU00, R&D Systems, USA). The levels of synaptophysin and PSD95 protein in the hippocampus were assessed using an ELISA kit for synaptophysin (CEA425Mu-1, Lifeome BioLabs, USA) and DLG4 / PSD95 ELISA Kit (LS-F7142-1, LifeSpan BioSciences, USA) according to the manufacturer’s instructions. Absorbance was measured at 450 nm with a reference wavelength of 450 nm via an Enspire^TM^ multilabel reader 2300 (PerkinElmer, Finland).

### Quantitation of TMAO and TMA levels

We added 80 μL of 80% acetonitrile to 20 μL of plasma for protein precipitation. As interior standards, _d9_-(trimethyl) TMAO and _d9_-(trimethyl) TMA were added to plasma samples. After 30 min, samples were centrifuged (20,000 × g, 4°C, 10 min) and then analyzed by stable isotope dilution liquid chromatography-tandem mass spectrometry (LC/MS), as described previously [60]. Briefly, LC/MS analysis was performed using an Agilent 6410 Triple Quad LC/MS (Agilent Technologies, USA). The capillary voltage was heated to 350 °C and set up at +4000 V. TMAO and TMA were examined using electrospray ionization in positive-ion mode with multiple reaction monitoring and characteristic production ion transitions: *m/z* 76-58, and *m/z* 60-44. TMAO and TMA levels in plasma were quantified using an Agilent 1260 Infinity HPLC system. Chromatographic separation was carried out on a hydrophilic interaction liquid chromatography (HILIC) column (150 × 2.1 mm, 3.5μm internal diameter, WATERS) shielded by a flex capillary HILIC guard column (10 × 2.1 mm, 3.5μm internal diameter, WATERS). The mobile phase (phase A: methanol with 0.1% formic acid, phase B: water with 0.1% formic acid) was used at a ratio of 30:70 with a flow rate of 0.20 ml/min. The calibration curves were generated by adding various concentrations of TMAO and TMA standards to control plasma, which allowed us to quantify plasma TMAO and TMA concentrations.

### Determination of the enzymatic activity of hepatic FMO

The activity of the FMO enzyme in the liver was measured as described [60], with minor alterations. Concisely, 100 mM nicotinamide adenine dinucleotide phosphate (NADPH), 100μM TMA, and 1 mg hepatic protein homogenate were blended in 250 μl reaction in 10 mM HEPES buffer (pH 7.4). The reaction was terminated by adding 0.2 N formic acid after eight hours, subsequently filtering using a 3K spin filter. The mixture was then instantly stored at -80 °C. For assays, the inner standard with the thawed filtrate was injected into LC/MS to quantify the oxidized product TMAO, as mentioned above.

### Multiplex bead analysis

The hippocampal supernatants were diluted at a 1:2 ratio in assay buffer and analyzed using a Luminex 200 (Luminex, USA). The cytokine expressions of interleukin-1β (IL-1β), IL-2, IL-4, IL-5, IL-6, IL-17, granulocyte colony-stimulating factor (G-CSF), interferon-induced protein 10 (IP-10), tumor necrosis factor α (TNF-α), interferon-γ (IFNγ), granulocyte-macrophage colony-stimulating factor (GM-CSF), regulated upon activation normal T cell expressed and secreted factor (RANTES), macrophage inflammatory protein-1β (MIP-1β), monocyte chemotactic protein-1 (MCP-1), Eotaxin were measured using a multiplex map kit (MCYTOMAG-70K, Millipore, USA).

### Metagenomic analyses

All feces were freely excreted by mice and then immediately collected. A total of 200-240 mg of fresh feces were collected. Total genomic DNA was extracted using an EZNA DNA kit (Omega Bio-Tek, Norcross, USA), and the purity and concentration of the extracted DNA were respectively measured using a NanoDrop 2000 spectrophotometer (Thermo Scientific, USA) and TBS-380 mini-fluorometer (Turner Biosystems, USA). The paired-end library was constructed by using a TruSeqTM DNA Sample Prep kit (Illumina Inc., USA). Paired-end sequencing was performed on the Illumina HiSeq4000 platform (Illumina Inc., USA), utilizing HiSeq 3000/4000 PE Cluster and HiSeq 3000/4000 SBS kits, according to manufacturer instructions. Adapter sequences were stripped from the 3' and 5' ends of paired-end Illumina reads by SeqPrep. Low-quality reads were removed using Sickle. Metagenomics data were assembled by MEGAHIT [61]. Contigs with a length of over 300 bp were selected for final assembly and used for further gene prediction and annotation. Open reading frames (ORFs) from each assembled contig were predicted by MetaGene [62]. All predicted genes with a 95% sequence identity were clustered using CD-HIT [63] and then mapped to representative sequences with 95% identity using a short oligonucleotide analysis package [64]. Characteristic sequences of non-redundant gene catalogs were aligned to the NCBI NR database with an e-value cutoff of 1e^-5^ by the basic local alignment search tool (BLAST) [65] for taxonomic annotations. Clusters of probiotics were annotated for their representative sequences employing best-hit against a PROBIO database (http://bidd2.nus.edu.sg/probio/homepage.htm) and with an e-value cutoff of 1e^-5^.

### Statistical analyses

All data were expressed as mean ± S.D. GraphPad Prism 8.0 was utilized to plot and analyze part data. Data from the two groups were compared by Student's *t*-tests. Comparison of data between multiple groups was performed using a one-way analysis of variance (ANOVA) followed by Dunnett’s *post hoc* test or a two-way repeated-measures ANOVA with *post-hoc* Tukey multiple comparisons test. Spearman correlation coefficients (R, v3.1.2) were used to measure correlations between TMAO levels in plasma and cognitive performance/pathology index. Receiver operating characteristic (ROC) curves were plotted and area under the curve (AUC) values were computed using the ROCR package in R. Principal component analysis plots were also computed using R. Results were considered statistically significant when *P* <0.05.

### Ethical approval

Animal experiments were processed in accordance with the references in the Guide for the Care and Use of Laboratory Animals published by the National Institutes of Health and were approved by the Institutional Animal Care and Use Committee of Harbin medical university.

## Supplementary Material

Supplementary Figures
