# Supplementary material for: Concomitant memantine and Lactobacillus plantarum treatment attenuates cognitive impairments in APP/PS1 mice
Source: Aging (Albany NY). 2020 Jan 6;12(1):628–49. doi: 10.18632/aging.102645 (PMC6977692; doi:10.18632/aging.102645)
Supplement: Supplementary Figures [file aging-12-102645-s001..pdf]

SUPPLEMENTARY FIGURES

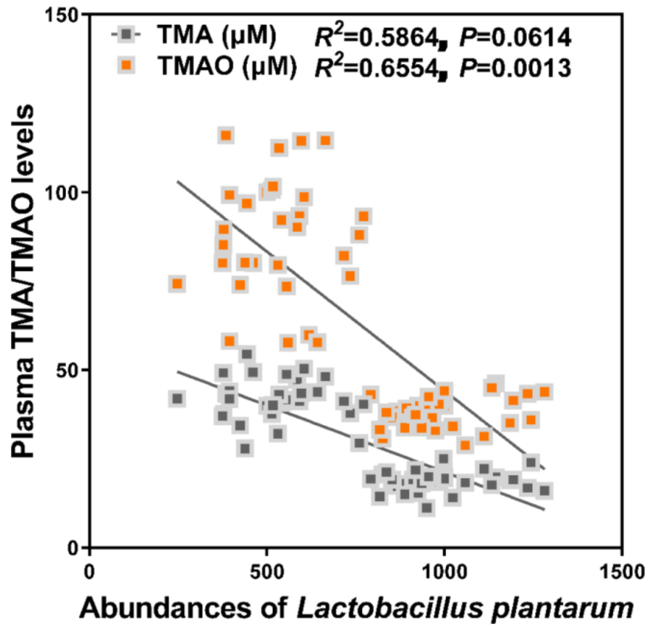

Supplementary Figure 1. Pearson correlation between the relative abundances of L. plantarum and plasma TMA and TMAO concentrations from mice grouped by dietary status (chow and choline). n=60.

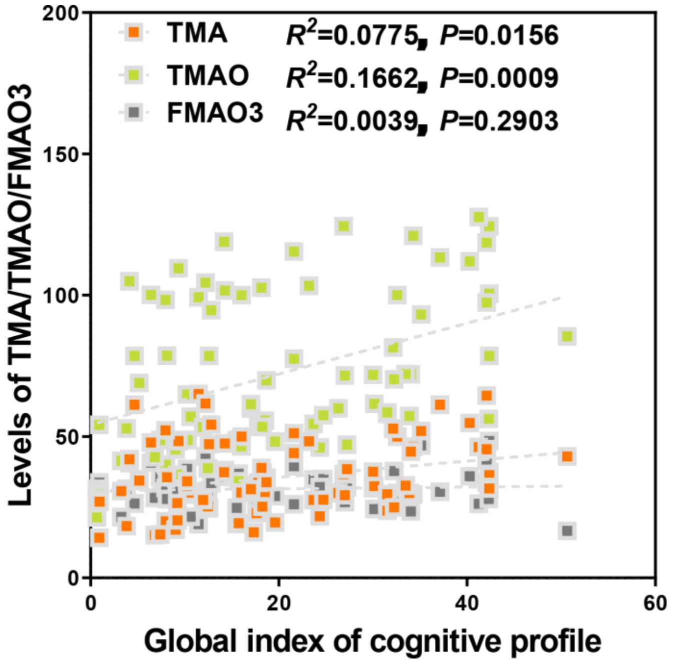

Supplementary Figure 2. Spearman correlation between the TMA/TMAO/FMAO3 concentrations and cognitive impairments in WT and APP/PS1 mice. n=75.

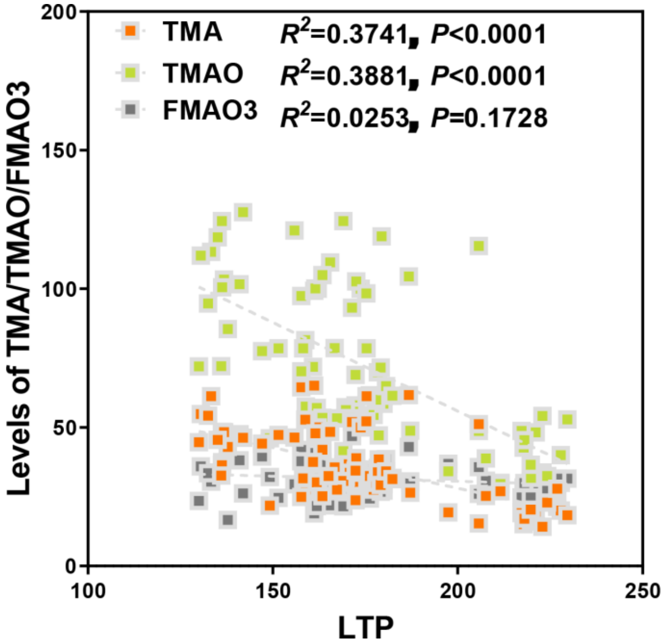

Supplementary Figure 3. Spearman correlation between the TMA/TMAO/FMAO3 concentrations and LTP in WT and APP/PS1 mice. LTP means long-term potentiation. n=75.

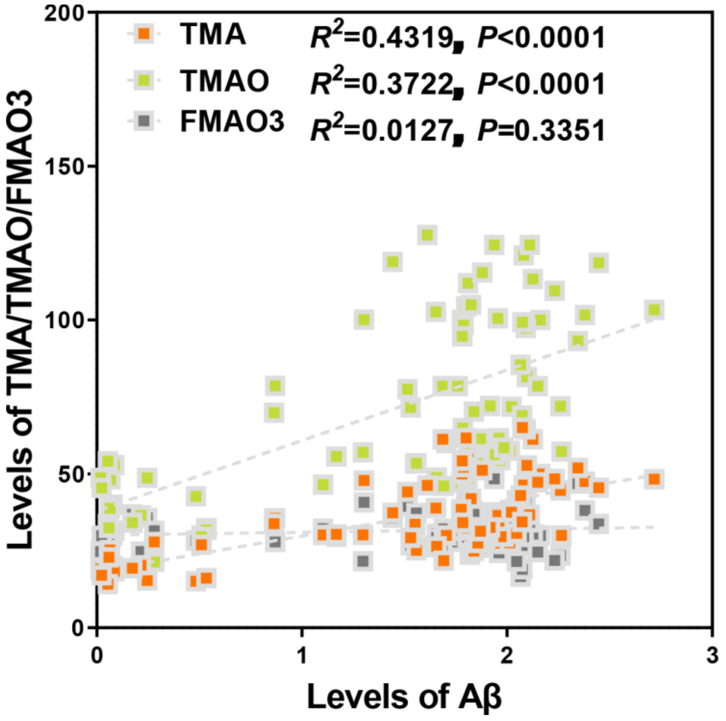

Supplementary Figure 4. Spearman correlation between the TMA/TMAO/FMAO3 concentrations and Aβ deterioration in the hippocampus of WT and APP/PS1 mice. n=75.

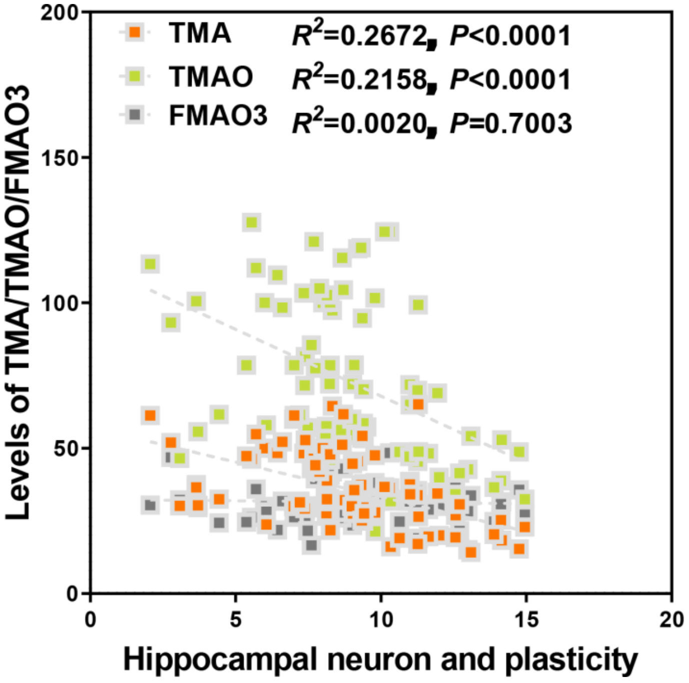

Supplementary Figure 5. Spearman correlation between the TMA/TMAO/FMAO3 concentrations and hippocampal neuron and plasticity deterioration in the hippocampus of WT and APP/PS1 mice. n=75.
